# Ion channels and atrial fibrillation: mitophagy as a key mediator

**DOI:** 10.3389/fphys.2025.1687578

**Published:** 2025-11-20

**Authors:** Xize Wu, Xiaorui Yan, Ruoxi Ma, Qiuying Wu, Xue Pan, Qihua Wu, Jiaqi Ren, Yuxi Huang, Shan Gao, Yue Li, Lihong Gong

**Affiliations:** 1 The First Clinical College, Liaoning University of Traditional Chinese Medicine, Shenyang, Liaoning, China; 2 Innovation engineering technology center of Traditional Chinese Medicine, Liaoning University of Traditional Chinese Medicine, Shenyang, Liaoning, China; 3 College of Traditional Chinese Medicine, Dazhou Vocational College of Chinese Medicine, Dazhou, Sichuan, China; 4 Department of Cardiology, Affiliated Hospital of Liaoning University of Traditional Chinese Medicine, Shenyang, Liaoning, China

**Keywords:** atrial fibrillation, mitophagy, ion channels, bioinformatics, mitochondrion

## Abstract

**Background:**

The prevalence of atrial fibrillation (AF) is increasing due to the aging population. Mitophagy is crucial for maintaining cardiomyocyte function, while ion channels play a key role in cardiac electrical activity. Dysfunction of ion channels can trigger AF. However, the role of mitophagy-related ion channel genes in AF remains unclear.

**Methods:**

AF-related datasets GSE41177 and GSE79768 were merged and batch-corrected for differential expression analysis. Mitophagy-related and ion channel-related genes were obtained from the MsigDB and GeneCards databases. Immune infiltration and functional enrichment analyses were performed. Sixty-five machine learning models were developed to identify Hub genes, with the optimal model selected based on receiver operating characteristic curves, F1 scores, and accuracy. An acute electrical remodeling model of atrial tachyarrhythmia was established in Sprague-Dawley rats by administering a mixture of acetylcholine-calcium chloride for 7 days. Hematoxylin-eosin, Masson, and Sirius red staining were used to detect histopathologic changes in the atrial myocardium. The expression of AF-related mitophagy ion channel genes and proteins was measured by qRT-PCR and Western blotting.

**Results:**

A total of 444 differentially expressed genes in AF were identified, and 9 AF-related mitophagy ion channel genes (AFRMICGs) were obtained (BAX, CTNNB1, DPYSL2, EPHX1, GLUL, GNB2, MIF, MYC, TLR4). Functional enrichment analysis indicated that the pathogenesis of AF is related to inflammation, immune response, ion channels, apoptosis, and various organelles and is associated with the PI3K/AKT, NF-kappa B, JAK-STAT, and mTOR pathways. Immune infiltration analysis showed higher resting dendritic cells and neutrophils and lower follicular helper T cells, M2 macrophages, and activated dendritic cells in AF patients. The glmBoost + Lasso model identified 4 Hub genes: BAX, GLUL, MIF, and TLR4. *In vivo* experiments showed disordered myocardial cell arrangement, collagen fiber proliferation, interstitial widening, fibrous septa formation, and uneven cytoplasmic staining. qRT-PCR results showed upregulation of BAX, MIF, TLR4, SLC8A1, and CaMKII genes, while the expression of Nav1.5, Kv1.5, hERG, Cav1.2, Cav1.3, Cav3.2, PINK1, Parkin, FUNDC1, BNIP3, NIX, MAP1LC3A, and MAP1LC3B genes was downregulated. Western blotting confirmed increased protein expression of BAX, MIF, and TLR4, whereas GLUL expression showed no significant difference at either the gene or protein level.

**Conclusion:**

BAX, MIF, and TLR4 are key genes linking mitophagy and ion channels in AF, which appear to influence the immune microenvironment by modulating immune cell infiltration.

## 1 Introduction

Atrial fibrillation (AF) is a common arrhythmia in clinical practice that often leads to severe complications such as heart failure, myocardial infarction, and stroke. It is associated with increased mortality and a significantly reduced quality of life (Hu et al., 2023; Ruddox et al., 2017). Recent epidemiologic studies confirm that AF remains a significant global public health problem, with notable regional and substantial variations. Over the past 31 years (1990–2021), the global prevalence of AF increased by 1.37-fold, and its incidence rose by 1.24-fold, with 4.48 million new cases reported in 2021, bringing the total number of prevalent cases to 52.55 million (Cheng et al., 2024). Current treatments for AF include risk factor control, medications for rate and rhythm control, and anticoagulation. For refractory cases, interventional procedures like cardiac radiofrequency ablation are used. However, these treatments have limitations, including adverse effects such as bleeding and a significant risk of AF recurrence (Wolfes et al., 2025). Thus, further elucidating the mechanisms of AF development and identifying precise intervention targets are urgently needed.

The pathogenesis of AF has not been fully elucidated, but the core pathological basis for its development and maintenance primarily involves two major mechanisms: atrial electrical remodeling and structural remodeling (Arabia et al., 2024). Electrical remodeling is mainly manifested as abnormal ion channel function in atrial myocytes, resulting in a shortening of action potential duration and increased dispersion of the effective refractory period. This creates a substrate for reentrant arrhythmias. Structural remodeling, on the other hand, involves morphological changes such as atrial fibrosis, myocardial hypertrophy, and dilation, which further promote the persistence and stabilization of AF (Andrade et al., 2014; Nattel and Harada, 2014) Recent studies have confirmed that mitochondrial dysfunction is a central hub driving these remodeling processes. As the energy factories of the cell, mitochondria generate adenosine triphosphate (ATP) through oxidative phosphorylation, providing the necessary energy for sustained contraction, ion pump operation, and electrical signaling in cardiomyocytes. In the AF state, atrial myocytes are subjected to rapid, disorganized, high-frequency electrical excitation. The dramatic increase in energy demand leads to mitochondrial overload and accelerates mitochondrial senescence and damage (Harada et al., 2017; Kohlhaas et al., 2017).

Mitochondrial dysfunction affects intracellular ionic homeostasis and membrane excitability through dual disruptions of energy crisis (ATP insufficiency) and oxidative stress (reactive oxygen species burst). These disruptions directly impair cardiomyocyte ion channel function and expression, driving the onset and progression of AF (Yang et al., 2014; Mason et al., 2020). Insufficient ATP production alters the function of all ATP-dependent enzymes and ion-transport proteins, thereby disrupting normal cellular excitability. Specifically, inhibition of the sodium-potassium pump (Na+/K + -ATPase) leads to an increase in intracellular Na + concentration. This activates the sodium-calcium exchanger in reverse mode, promoting Ca2+ influx and triggering calcium overload (Chung et al., 2024; Chkadua et al., 2024). Simultaneously, ATP deficiency impairs the function of sarcoplasmic/endoplasmic reticulum Ca2+-ATPase (SERCA2a), hindering cytoplasmic calcium reuptake into the SR and further exacerbating calcium overload.

Excess reactive oxygen species (ROS) also play a critical role in this process. They directly cause oxidative modification of key ion channel proteins. Oxidation of ryanodine receptor 2 (RyR2) and inhibition of SERCA activity lead to SR calcium leakage, amplifying calcium overload10. Additionally, ROS inhibit potassium channel function and decrease the expression of Kv currents (IKr, IKs, and IKur), resulting in delayed repolarization, prolonged action potential duration, and a shortened effective refractory period (Cerbai et al., 1991; Li et al., 2008; Nerbonne and Kass, 2005). The increase in the late sodium current (late INa) leads to prolonged action potential duration and increased early afterdepolarizations and aftercontractions, which in turn elevate intracellular Na+ and Ca2+ concentrations, ultimately resulting in electrical and contractile dysfunction (Song et al., 2006). Moreover, high levels of ROS interfere with excitation-contraction coupling, induce cardiac adaptive remodeling via redox-sensitive kinases, and trigger cell death through mitochondrial permeability transition (Bertero and Maack, 2018). Thus, mitochondrial dysfunction is a critical driver in the development of AF, and maintaining mitochondrial functional homeostasis is essential for the prevention and treatment of AF.

Mitophagy, a key mechanism for mitochondrial quality control, selectively removes damaged mitochondria to prevent ROS accumulation and preserve the healthy mitochondrial network. However, chronic AF-related stress (e.g., calcium overload, sustained ROS exposure) can impair mitophagy pathways, resulting in the accumulation of dysfunctional mitochondria (Lin et al., 2024a; Zhu et al., 2022; Sang et al., 2024). Moderate mitophagy maintains cardiomyocyte calcium homeostasis and energy balance, exerting cardioprotective effects (Zhou et al., 2020). Defective mitophagy profoundly affects ion channel function and expression by exacerbating oxidative stress and disrupting calcium homeostasis. This defect constitutes a core pathological link connecting mitochondrial dysfunction to the vicious cycle of AF self-perpetuation (“AF begets AF”). Targeted enhancement of mitophagy or improvement of mitochondrial quality may represent a novel strategy to intervene in the electrical remodeling of AF (Mauriello et al., 2024).

This study combined bioinformatics analysis and experimental validation to uncover key genes and molecular networks underlying the interaction between mitophagy and ion channels in AF. The objective was to elucidate the molecular mechanisms underlying the “mitophagy defects → ion channel dysfunction → electrical remodeling” axis. First, AF-related mitophagy-ion channel genes (AFRMICGs) were identified using differential expression analysis. Functional enrichment analysis was then performed on these AFRMICGs to elucidate their functional characteristics, and immune infiltration analysis was employed to assess their associated immune microenvironment features. Subsequently, multiple machine learning algorithms were applied to identify and characterize Hub gene candidates. Subsequently, an acute electrically remodeled rat model of atrial tachyarrhythmia was constructed to verify, at the *in vivo* level, mitophagy activity, key ion channel expression/function, and expression changes of the Hub gene candidates. Finally, based on the identified Hub genes, an AF risk prediction nomogram was constructed, and their upstream regulatory networks were thoroughly analyzed, including transcription factor regulation and competitive endogenous RNA (ceRNA) interactions. As gene therapy offers a promising approach to modulate AF’s molecular drivers (O'Donnell et al., 2025), this study is expected to identify potential targets for the development of novel antiarrhythmic drugs acting via the mitophagy pathway.

## 2 Materials & methods

### 2.1 Bioinformatics analysis

#### 2.1.1 Subjects and dataset acquisition

**FIGURE 1:**
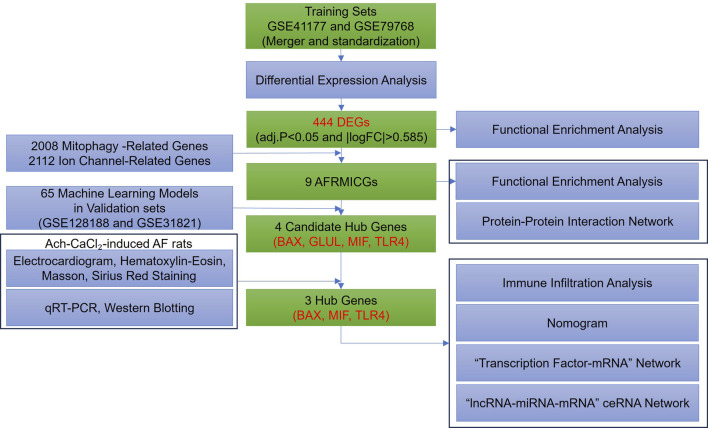
Flowchart of this study.

The overall study design is illustrated in Figure 1. Four gene expression profiles related to AF were retrieved from the Gene Expression Omnibus (GEO, https://www.ncbi.nlm.nih.gov/geo/) database using the keywords “atrial fibrillation” (GSE41177, GSE79768, GSE128188, and GSE31821), with the search limited to “*Homo sapiens*” and the tissue type restricted to left atrial appendage. The GSE41177 dataset (GPL570) comprises left atrial appendage tissues from 6 normal individuals and 32 AF patients, resulting in a total of 38 cases (Yeh et al., 2013); the GSE79768 dataset (GPL570) includes left atrial appendage tissues from 6 normal individuals and 7 AF patients, resulting in a total of 13 cases (Tsai et al., 2016); the GSE128188 dataset (GPL18573) contains left atrial appendage tissues from 5 normal individuals and 5 AF patients, resulting in a total of 10 cases (Thomas et al., 2019); and the GSE31821 dataset (GPL570) consists of left atrial appendage tissues from 2 normal individuals and 4 AF patients, resulting in a total of 6 cases. The GSE41177 and GSE79768 datasets were merged and standardized to form the training set, while GSE128188 and GSE31821 served as the validation set for machine learning.

Mitophagy-related genes were obtained from the Molecular Signature Database (MsigDB, https://www.gsea-msigdb.org/) and augmented with genes that had a “relevance score” greater than the mean (0.9267) from the GeneCards (https://www.genecards.org/, accessed on 20 May 2025) database under the keyword “mitophagy.”

Ion channel-related genes were obtained from the GeneCards (accessed on 15 March 2025) database and filtered to include only genes with a “relevance score” greater than the average (3.3251).

#### 2.1.2 Identification of differentially expressed genes (DEGs)

The GSE41177 and GSE79768 datasets were merged and standardized using the “Affy” R package. To address batch effects arising from different platforms and studies, cross-platform batch effect correction was performed using the ComBat algorithm from the “SVA” R package. A model matrix incorporating the biological condition (disease vs. control) was included as a covariate to preserve biological variance while removing technical artifacts. The success of batch effect removal was confirmed by the clear separation of samples by disease state rather than by dataset origin in the post-correction principal component analysis plot. The merged dataset was used to identify AF-related DEGs by comparing the disease and control groups using the “limma” R package with a linear modeling approach, with the criteria for DEG selection set at adj. *P* < 0.05 and |log_2_FC|>0.585 (approximately equivalent to a 1.5-fold change) (Ritchie et al., 2015).

#### 2.1.3 Functional enrichment analysis

Genes were imported into the STRING (https://cn.string-db.org/, accessed on 20 May 2025) database to construct a medium-confidence protein-protein interaction network. Gene Ontology and Kyoto Encyclopedia of Genes and Genomes analyses were performed using the DAVID (https://davidbioinformatics.nih.gov/home.jsp, accessed on 20 May 2025) database. Gene Set Enrichment Analysis (GSEA) was conducted using the “GSEA” R package (Subramanian et al., 2005). Kyoto Encyclopedia of Genes and Genomes pathway gene sets (c2. cp.kegg.v7.4. symbols.gmt) were obtained from the MSigDB database. Genes were ranked in descending order based on the logFC values of differentially expressed genes. Multiple hypothesis testing corrections were applied using the Benjamini-Hochberg method, and significant pathways with an FDR<0.05 were retained. The top 5 most significantly enriched pathways (normalized enrichment score>0 for upregulated) were visualized using the enrichplot.

#### 2.1.4 Immune infiltration and correlation analysis

The degree of infiltration of 22 immune cells was quantified using the CIBERSORT deconvolution algorithm with the LM22 signature matrix (Chen et al., 2018). Data preprocessing involved automatic logarithmic transformation, quantile normalization, and Z-score normalization. The ν-support vector regression (ν-SVR) algorithm was implemented with parameter optimization (ν = 0.25–0.75), model selection by root-mean-square error minimization, and non-negative constraint enforcement. To evaluate the reliability of our results, we performed a 1000-permutation test (perm = 1,000), retaining significant results with a *P* < 0.05. Differences between the two groups were compared using the Wilcox test, and the results were visualized using the “vioplot” R package (Hu, 2020). Subsequently, Spearman correlation analysis was employed to reveal the relationship between AFRMICGs and immune cells.

#### 2.1.5 Machine learning methods

The “randomForestSRC”, “glmnet”, “plsRglm”, “gbm”, “caret”, “mboost”, “e1071”, “BART”, “MASS”, “snowfall”, and “xgboost” R packages were used to establish 65 machine learning models for screening Hub genes, including the least absolute shrinkage and selection operator (LASSO) regression, random forest (RF) model, support vector machine (SVM) model, gradient boosting with component-wise linear model (glmBoost), extreme gradient boosting (XGBoost), gradient boosting machine (GBM), elastic net (Enet), and stepglm, among others.

The merged dataset of GSE41177 and GSE79768 was used as the training set, and the GSE128188 and GSE31821 datasets were used as the validation set. To ensure robust performance estimation and mitigate overfitting, 10-fold cross-validation was repeated 5 times on the training set for hyperparameter optimization. Model performance was comprehensively evaluated using multiple metrics: area under the receiver operating characteristic (ROC) curve (AUC), F1-score, and accuracy. The final model selection prioritized optimal discrimination performance while maintaining parsimony in feature number. Hub genes were defined as features consistently selected by the top-performing models across validation cohorts. The predictive capacity of these Hub genes was further assessed in the independent validation sets using ROC analysis implemented with the “pROC” R package.

#### 2.1.6 Construction and validation of a nomogram model

A nomogram model incorporating the Hub genes identified from the machine learning process was established using the “rms” R package to predict the risk of developing AF, and its predictive power was evaluated by using calibration curves and decision curve analysis.

#### 2.1.7 “Transcription factor (TF)-mRNA” network

The TRRUST is a database focusing on the regulatory relationships between TFs and their target genes, which is built by extracting information from scientific literature in the PubMed database through text mining techniques to create a comprehensive transcriptional regulatory network (Han et al., 2018). Hub genes were imported into the TRRUST (https://www.grnpedia.org/trrust/, accessed on 20 May 2025) database to obtain their TF, with the species specified as “Human.” In addition, the TF of the Hub genes was also obtained from the hTFtarget (http://guolab.wchscu.cn/hTFtarget/, accessed on 20 May 2025) database (Zhang et al., 2020).

#### 2.1.8 “lncRNA-miRNA-mRNA” ceRNA interaction network

Four bioinformatics tools, TargetScan (http://www.targetscan.org/vert_72/, accessed on 20 May 2025), DIANA-microT (http://diana.imis.athena-innovation.gr/DianaTools/index.php, accessed on 20 May 2025), miRmap (https://mirmap.ezlab.org/, accessed on 20 May 2025), and TarBase (https://dianalab.e-ce.uth.gr/tarbasev9, accessed on 20 May 2025), were utilized to predict miRNAs targeting the Hub genes, and miRNAs predicted by at least three tools were selected. In addition, the predicted miRNAs were imported into the DIANA Tools-LncBase Experimental v.2 (https://dianalab.e-ce.uth.gr/html/diana/web/index.php?r=lncbasev2%2Findex-experimental, accessed on 20 May 2025) database to obtain their upstream lncRNAs. Finally, the ceRNA network was constructed.

#### 2.1.9 Statistical analysis

All bioinformatics statistical analyses were performed using R software, and a *P* < 0.05 was considered significant.

### 2.2 Experimental validation

#### 2.2.1 Animals, reagents, and instruments

Experimental animals: Forty Sprague-Dawley rats, 160–200 g, 6–8 weeks old, male, purchased from the Liaoning Changsheng Biotechnology Co. The experimental animals were fed and experimented on in the Animal Experiment Center of Liaoning University of Traditional Chinese Medicine with the following environmental parameters: temperature 22 °C–24 °C, humidity 40%–60%, free feeding and drinking, and 12h/12h day/night alternation.

Reagents: Acetylcholine Chloride (Ach) (Shanghai yuanye, Cat. S30170-5g); Anhydrous Calcium Chloride (CaCl_2_) (Shanghai yuanye, Cat. S24110-500 g); Isoflurane (SAMVET, Lot.235180101); Hematoxylin-Eosin Constant Dye Kit (Servicebio, Cat. G1076); Masson Dye Solution Set (Servicebio, Cat. G1006); Differentiation Fluid (Servicebio, Cat. G1039); Sirius Red Staining Solution (Servicebio, Cat. G1018); Environmentally Friendly Dewaxing Liquid (Servicebio, Cat. G1128); Paraformaldehyde Fixative (Neutral) (Servicebio, Cat. G1101); 75% ethanol (Shenyang GuangCai, Cat.20250102); Absolute ethanol (Sinopharm Chemical Reagent Co.,Ltd., Cat.100092683); Xylene (Sinopharm Chemical Reagent Co.,Ltd., Cat.10023418); Normal Butanol (Sinopharm Chemical Reagent Co.,Ltd., Cat.100052190); Neutral Gum (Sinopharm Chemical Reagent Co.,Ltd., Cat.10004160); RNA Extraction Solution (Servicebio, Cat. G3013); chloroform Substitute (Servicebio, Cat. G3014); Isopropanol (Sinopharm Chemical Reagent Co.,Ltd., Cat.80109218); SweScript All-in-One RT SuperMix for qPCR (Servicebio, Cat. G3337); 2×Universal Blue SYnthetic Binding Reagent Green qPCR Master Mix (Servicebio, Cat. G3326); Bicinchoninic Acid Protein Concentration Measurement Kit (Beyotime, P0010); Radioimmunoprecipitation Assay Lysate (Beyotime, P0013B); Sodium Dodecyl Sulfate Polyacrylamide Gel Electrophoresis (SDS-PAGE) Gel Rapid Preparation Kits (Beyotime, P0015L); Maker (Epizyme Biotech, Cat. WJ103); β-Actin Rabbit mAb (Abclonal, Cat. AC038); Anti-MIF (UpingBio, Cat. YP-Ab-15949); Anti-GLUL (UpingBio, Cat. YP-Ab-12846); Anti-TLR4 (UpingBio, Cat. YP-Ab-13172); Anti-BAX (BOSTER, Cat. A00183); Goat Anti-Rabbit IgG (H + L), HRP (UpingBio, Cat. YP848537-H).

Instruments: Basic Table Top Anesthesia Machine (Harvard Apparatus, TABLETOP); PowerLab 15T Multi-Conductive Physiological Recorder (ADInstruments, ML4818); Embedding Machine (Wuhan Junjie, JB-L5); Pathology Slicer (Leica, RM2016); Freezing Microtome (Thermo, CRYOSTAR NX50); Upright Optical Microscope (Nikon, ECLIPSEE100); Imaging System (Nikon, DS-U3); Fluorescent Quantitative PCR Instrument (Bio-rad, CFX Connect); PCR Instrument (Eastwin, ETC.,811); Dehydrator (DIAPATH, Donatello).

#### 2.2.2 Model development and evaluation

Rats were randomly assigned to two groups: a control group (n = 20) and a model group (n = 20). To establish a model of atrial tachyarrhythmia (encompassing atrial fibrillation and atrial flutter), the model group received daily tail vein injections of a mixture of Ach-CaCl_2_ (0.1 mL/100 g) for 7 days (Wu et al., 2025). Throughout the modeling period, the model group was fed a high-fat diet, whereas the control group received a normal diet along with daily tail vein injections of an equivalent volume of saline. Electrocardiograms were recorded after the modeling period. The successful induction of atrial tachyarrhythmia was primarily defined by the replacement of distinct P waves with rapid atrial oscillations (fibrillatory f-waves or flutter F-waves) accompanied by an irregular ventricular response. This study was conducted with approval from the Ethics Committee for Animal Experiments at Liaoning University of Traditional Chinese Medicine (No.21000042022075).

For euthanasia and tissue collection, rats were deeply anesthetized via inhalation of 2%–3% isoflurane for 4 min, followed by exsanguination via transection of the abdominal aorta. Subsequently, rats were fixed in a supine position, the abdominal cavity was opened to expose the heart, and the heart was then ligated, washed with saline, and fixed in 4% paraformaldehyde or frozen in liquid nitrogen. The paraformaldehyde-fixed atrial tissue was rinsed with phosphate-buffered saline. The tissue then underwent gradient dehydration (70%, 80%, 95%, and 100% ethanol for 1 h each, and xylene for transparency for 30 min), and 5 μm serial sections were prepared after paraffin embedding. Five rats were randomly selected from each of the control and model groups for histopathological staining.

#### 2.2.3 Electrocardiogram recording and analysis

The rat was fixed in a supine position on an operating board after anesthesia by securing the ankle joints of all four limbs with rubber bands. The skin on the inner side of the ankle joints was disinfected with 75% alcohol cotton balls. Needle electrodes were inserted subcutaneously into the right forelimb and left hindlimb, and connected to the negative and positive terminals of the four-channel polygraph physiological acquisition and analysis system, respectively, followed by connection of the ground wire. The LabChart Pro software was launched, and parameters were set as follows: Range (R): 500 μV, Low Pass (L): 50 Hz, High Pass (H): 0.5 Hz, horizontal expansion ratio of 5:1. This setup allowed for recording of the simulated standard limb lead II electrocardiogram. For rats in the control group, electrocardiogram acquisition began after the baseline stabilized. For rats in the model group, after the baseline stabilized, a segment of normal electrocardiogram was first recorded. Then, the Ach-CaCl_2_ mixture was injected via the tail vein. Recording continued once the typical electrocardiogram pattern appeared and was stopped after the typical electrocardiogram pattern disappeared. Furthermore, the atrial rate was quantified by measuring the mean F-F or f-f interval during the arrhythmia and compared to the P-P interval during sinus rhythm.

#### 2.2.4 Hematoxylin-eosin staining

The paraffin sections underwent sequential dewaxing in environmentally friendly Dewaxing Liquids I and II for 20 min each, followed by 5-min treatments in Anhydrous ethanol I and II and 75% ethyl alcohol. Frozen sections were equilibrated to room temperature, fixed with tissue fixative for 15 min, and rinsed with running water. For hematoxylin staining, sections were stained for 3–5 min, differentiated, and rinsed. This was followed by a brief rinse in 95% ethanol and a 15-s counterstain with eosin dye. Dehydration was achieved with 2-min treatments in absolute ethanol (I, II, III), normal butanol (I, II), and xylene (I, II), and sections were finally sealed in neutral gum.

#### 2.2.5 Masson staining

The paraffin sections were dewaxed in environmentally friendly Dewaxing Liquid I for 20 min, followed by Liquid II for another 20 min. The sections were then treated with anhydrous ethanol I and II for 5 min each and 75% ethyl alcohol for 5 min, with thorough tap water rinses after each step. Frozen sections were equilibrated to room temperature after removal from −20 °C, fixed with tissue fixative for 15 min, and rinsed with running water. The slices were soaked in Masson A overnight and rinsed. Masson B and C were mixed 1:1 to prepare the staining solution. Sections were stained for 1 min, differentiated with 1% hydrochloric acid alcohol for several seconds, and rinsed. They were then soaked in Masson D for 6 min and rinsed again. After a 1-min treatment with Masson E, slides were drained and placed in Masson F for 2–30 s without washing. After rinsing with 1% glacial acetic acid, the sections were dehydrated with anhydrous ethanol. Finally, slides were cleared in 100% ethanol for 5 min and xylene for 5 min and sealed with neutral gum. The collagen volume fraction was then calculated, defined as (collagen area)/(total area) × 100%.

#### 2.2.6 Sirius Red staining

The paraffin sections were dewaxed in environmentally friendly Dewaxing Liquids I and II for 20 min each, then in anhydrous ethanol for 5 min each, followed by a 5-min treatment in 75% ethyl alcohol. Frozen sections were brought to room temperature, fixed with tissue fixative for 15 min, and rinsed with running water. Slides were stained with Sirius Red for 8 min and dehydrated with anhydrous ethanol. Sections were then cleared in xylene for 5 min and sealed with neutral gum. The percentage of Sirius red staining area was then calculated, defined as (Sirius red area)/(total area) × 100%.

#### 2.2.7 Quantitative Real-Time Polymerase Chain Reaction (qRT-PCR)

Total RNA was extracted from atrial tissue samples of 5 randomly selected normal and 5 model rats, with each rat representing an independent biological replicate (n = 5 per group). The RNA was purified through treatment with a chloroform substitute, precipitation with isopropanol, and washing with ethanol. The purified RNA was then reverse transcribed into cDNA. Quantitative PCR analysis was conducted using SYBR Green qPCR Master Mix and specific primers. Each cDNA sample was run in triplicate (three technical replicates). The cycle threshold (Ct) values from the three technical replicates were averaged for each biological replicate. Relative gene expression changes were then calculated for each of the 5 biological replicates per group using the 2^(-^ΔΔ^CT) method, normalized to an internal reference gene. The reagents used in the experiment were provided by Servicebio (Wuhan) Co., Ltd. (Table 1).

**TABLE 1:** A list of the primers used in the qRT-PCR.

Type	Gene	Primer sequence (5′-3′)	Bp
Internal reference	GAPDH	Forword: CTGGAGAAACCTGCCAAGTATG	138
		Reverse: GGTGGAAGAATGGGAGTTGCT	
Hub genes	BAX	Forword: GGGTGGTTGCCCTTTTCTACTT	104
		Reverse: GAAGTCCAGTGTCCAGCCCAT	
	GLUL	Forword: GAAGGGCTACGCTGCAAGA	126
		Reverse: GAGGTACATGTCGCTGTTGGA	
	MIF	Forword: GGACCGGGTCTACATCAACTATTA	144
		Reverse: GGTGGATAAACACAGAACGGG	
	TLR4	Forword: CCAGGTGTGAAATTGAGACAATTG	191
		Reverse: AAGCTGTCCAATATGGAAACCC	
Mitophagy	PINK1	Forword: TGCAATGCCGCTGTGTATGA	113
		Reverse: TCTGCTCCCTTTGAGACGAC	
	PARKIN	Forword: GGAAGTGGTTGCTAAGCGACAG	173
		Reverse: CTCCAGAGGCATTTGTTTCGT	
	FUNDC1	Forword: GGCTGGTGTGCAGGATTTTTAT	231
		Reverse: CTGCTTGATAAAGTCTGTTGCTT	
	BNIP3	Forword: CTTCAGCAATGGGAATGGGAG	160
		Reverse: GGTATCTTGTGGTGTCTGGGAGC	
	NIX	Forword: GGCAACGGTAATGGAAATGG	165
		Reverse: TTCTTGTGGTGAAGGGCTGTC	
	MAP1LC3A/LC3A	Forword: AGGTGCAGCAGATCCGTGA	266
		Reverse: TCCGTCTTCATCCTTCTCCTGTT	
	MAP1LC3B/LC3B	Forword: GCTCAATGCTAACCAAGCCT	108
		Reverse: AAGCCGTCTTCATCTCTCTCG	
Sodium channel	SCN5A/Nav1.5	Forword: CCATAGTGTCGGTCCTGGTCAT	178
		Reverse: ACCGTAGGGCCAGAGGAAAGT	
	SLC8A1/NCX1	Forword: AGGGGAGGACTTTGAGGACA	155
		Reverse: CACTCATCTCCACCAGACGG	
Potassium channel	KCNA5/Kv1.5	Forword: CCATAGTGTCGGTCCTGGTCAT	178
		Reverse: ACCGTAGGGCCAGAGGAAAGT	
	KCNH2/hERG	Forword: GCTGCTACAGAGGCAAATGACC	143
		Reverse: GGGAAACCTGAGAAAGCGAG	
Calcium Channel	CACNA1C/Cav1.2	Forword: AAGCACGTCGTTCAGTGTGT	220
		Reverse: GAGGCTGGATAATGGGGTGG	
	CACNA1G/Cav3.1	Forword: CGTGGTTCGAGCGAGTCAGTA	154
		Reverse: ATTTCCACAGCAAAGAAGGCA	
	CACNA1H/Cav3.2	Forword: CTACTACTGCGAGGGCACAG	180
		Reverse: GCATCCAGCCCGTCATACAT	
	CaMKII	Forword: CAAACTACTGTAATCCACAACCCTG	162

#### 2.2.8 Western blotting

Total protein was extracted from atrial tissue samples using pre-chilled radioimmunoprecipitation assay lysis buffer containing protease inhibitors. Protein concentration was determined by a bicinchoninic acid assay. For each sample, proteins (40 μg per lane) were separated by sodium dodecyl sulfate-polyacrylamide gel electrophoresis on 10% gels and then electrophoretically transferred to polyvinylidene fluoride membranes. After transfer, the membranes were blocked and subsequently incubated with diluted primary antibodies (anti-MIF, anti-GLUL, anti-TLR4, anti-BAX) at 4 °C overnight. Membranes were then incubated with species-matched horseradish peroxidase-conjugated secondary antibodies. Protein bands were visualized using an enhanced chemiluminescence detection system. This analysis included three randomly selected biological replicates from both the control and AF groups (n = 3 per group), with each sample processed individually without technical replicates. The relative intensity of each protein band was quantified using ImageJ software and normalized to a loading control (Table 2).

**TABLE 2:** Antibodies and reagents used for western blot analysis.

Reagents	Host species	Vendor	Catalog number	Working concentration
Maker	-	Epizyme biotech	WJ103	-
MIF	Rabbit	UpingBio	YP-Ab-15949	1:1,000
GLUL	Rabbit	UpingBio	YP-Ab-12846	1:1,000
TLR4	Rabbit	UpingBio	YP-Ab-13172	1:1,000
BAX	Rabbit	BOSTER	A00183	1:1,000
β-actin	Rabbit	Abclonal	AC038	1:4,000
Goat Anti-Rabbit IgG (H + L), HRP	Goat	UpingBio	YP848537-H	1:8,000
Radioimmunoprecipitation assay lysis buffer	-	Beyotime	P0013B	-
Bicinchoninic acid assay kit	-	Beyotime	P0010	-
SDS-PAGE gel kit	-	Beyotime	P0015L	-

#### 2.2.9 Statistical analysis

Data are presented as the mean ± standard deviation. Statistical analyses were performed using GraphPad Prism software 9.0. The normality of data distribution was assessed using the Shapiro-Wilk test, and the homogeneity of variances was verified by the Brown-Forsythe test. For multiple group comparisons that satisfied both assumptions, one-way analysis of variance (ANOVA) was applied, followed by Tukey’s honestly significant difference (HSD) post hoc test for specific comparisons. When parametric assumptions were violated, the non-parametric Kruskal-Wallis test was used instead. A two-tailed P-value of less than 0.05 was considered statistically significant (Table 3).

**TABLE 3:** List of abbreviations.

Abbreviation	Definition
Ach	Acetylcholine Chloride
AF	Atrial Fibrillation
AFRMICGs	Atrial Fibrillation-Related Mitophagy Ion Channel Genes
AKT	Protein Kinase B
ATP	Adenosine Triphosphate
AUC	Area Under the Curve
BAX	BCL-2-Associated X Protein
BNIP3	BCL2/adenovirus E1B 19 kDa Protein-Interacting Protein 3
CaCl2	Calcium Chloride
CACNA1C/Cav1.2	Voltage-Dependent L-Type Calcium Channel Subunit Alpha-1C
CACNA1G/Cav3.1	Voltage-Dependent T-Type Calcium Channel Subunit Alpha-1G
CACNA1H/Cav3.2	Voltage-Dependent T-Type Calcium Channel Subunit Alpha-1H
CaMKII	Calcium/Calmodulin-Dependent Protein Kinase II
ceRNA	Competitive Endogenous RNA
CTNNB1	Catenin Beta-1
DEGs	Differentially Expressed Genes
DPYSL2	Dihydropyrimidinase-Related Protein 2
Enet	Elastic Net
EPHX1	Epoxide Hydrolase 1
FUNDC1	FUN14 Domain-Containing Protein 1
GBM	Gradient Boosting Machine
GEO	Gene Expression Omnibus
GLUL	Glutamine Synthetase
GNB2	Guanine Nucleotide-Binding Protein G(I)/G(S)/G(T) Subunit Beta-2
GSEA	Gene Set Enrichment Analysis
KCNA5/Kv1.5	Potassium Voltage-Gated Channel Subfamily A Member 5
KCNH2/hERG	Potassium Voltage-Gated Channel Subfamily H Member 2
LASSO	Least Absolute Shrinkage and Selection Operator
MAP1LC3A/LC3A	Microtubule-Associated Protein 1 light Chain 3 Alpha
MAP1LC3B/LC3B	Microtubule-Associated Protein 1 light Chain 3 Beta
MIF	Macrophage Migration Inhibitory Factor
MsigDB	Molecular Signature Database
mTOR	Mammalian Target of Rapamycin
MYC	Myc Proto-Oncogene Protein
NIX	BCL2/Adenovirus E1B 19 kDa Protein-Interacting Protein 3-Like
NLRP3	NOD-Like Receptor Thermal Protein Domain Associated Protein 3
PARKIN	E3 Ubiquitin-Protein Ligase Parkin
PI3K	Phosphoinositide 3-Kinase
PINK1	Serine/threonine-Protein Kinase PINK1, Mitochondrial
qRT-PCR	Quantitative Real-Time Polymerase Chain Reaction
RF	Random Forest
ROS	Reactive Oxygen Species
RyR2	Ryanodine Receptor 2
SCN5A/Nav1.5	Sodium Channel Protein Type 5 Subunit Alpha
SDS-PAGE	Sodium Dodecyl Sulfate Polyacrylamide Gel Electrophoresis
SERCA2a	Sarcoplasmic/Endoplasmic Reticulum Ca²⁺ ATPase 2a
SLC8A1/NCX1	Sodium/Calcium Exchanger 1
SR	Sarcoplasmic Reticulum
SVM	Support Vector Machine
TF	Transcription Factor
TLR4	Toll-like receptor 4
XGBoost	Extreme Gradient Boosting

## 3 Results

### 3.1 The pathogenesis of AF may be closely related to inflammation, immune response, ion channels, apoptosis, and various cellular organelles

**FIGURE 2:**
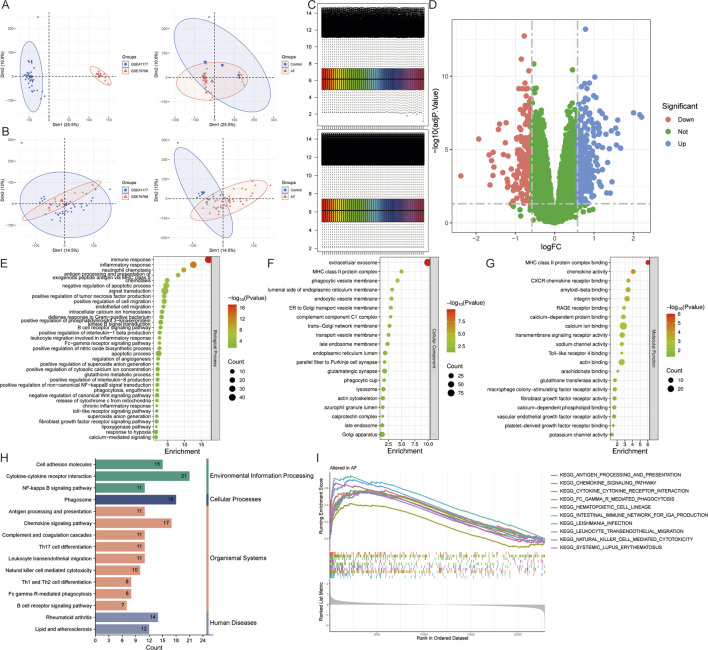
Identification and functional enrichment analysis of DEGs in atrial fibrillation (AF). **(A,B)** Principal component analysis of AF-related datasets GSE41177 and GSE79768 **(A)** before and **(B)** after merging. **(C)** Heatmap of gene expression levels before and after standardization of the merged dataset. **(D)** Volcano plot for DEGs. **(E–I)** The **(E)** biological process, **(F)** cellular component, **(G)** molecular function, **(H)** pathway, and **(I)** Gene Set Enrichment Analysis of all DEGs.

After merging (Figures 2A,B) and batch-effect correction of (Figure 2C) the AF-related datasets GSE41177 and GSE79768, differential expression analysis of the combined data identified 444 DEGs, including 258 upregulated genes and 186 downregulated genes (adj.P < 0.05 and |logFC|>0.585) (Figure 2D). Functional enrichment analysis of all DEGs revealed 174 biological processes, 58 cellular components, 62 molecular functions, and 43 pathways (P < 0.05). The biological processes were mainly enriched in immune response (defense response to Gram-positive bacteria, B-cell receptor signaling pathway), inflammatory response (production of tumor necrosis factor, interleukin-1 beta, and interleukin 8 production), cell chemotaxis (neutrophil chemotaxis, endothelial cell migration, leukocyte migration involved in inflammatory response), apoptosis (release of cytochrome c from mitochondria), signaling (calcium-mediated signaling), calcium homeostasis (intracellular calcium ion homeostasis), and oxidative stress (positive regulation of superoxide anion generation, glutathione metabolic process, superoxide anion generation) (Figure 2E). The significant cellular components included extracellular exosome, lumenal side of endoplasmic reticulum membrane, ER to Golgi transport vesicle membrane, late endosome membrane, endoplasmic reticulum lumen, parallel fiber to Purkinje cell synapse, glutamatergic synapse, lysosome, calprotectin complex, and Golgi apparatus (Figure 2F). The significant molecular functions included ion binding (calcium-dependent protein binding, calcium ion binding, sodium channel activity, potassium channel activity), arachidonate binding, glutathione transferase activity, and macrophage colony-stimulating factor receptor activity (Figure 2G). The significantly enriched signaling pathways included various diseases (Rheumatoid arthritis, Lipid and atherosclerosis), immune responses (antigen processing and presentation, Th17 cell differentiation, natural killer cell mediated cytotoxicity, B cell receptor and T cell receptor signaling pathway), inflammatory responses (leukocyte transendothelial migration), NF-kappa B, JAK-STAT, and mTOR signaling pathway (Figures 2H,I). The above results suggest that the pathogenesis of AF is closely related to inflammatory response, immune response, ion channels, oxidative stress, apoptosis, and various cellular organelles.

### 3.2 Mitophagy ion channel-related genes alter the immune infiltration microenvironment in AF patients

**FIGURE 3:**
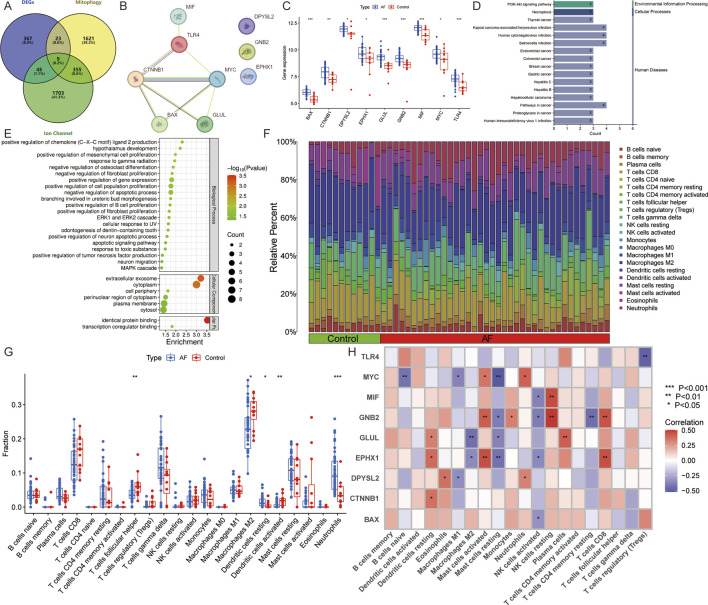
Identification of AF-related mitophagy-ion channel genes (AFRMICGs) and immune infiltration analysis. **(A)** Venn diagram showing 9 AFRMICGs. **(B)** Protein-protein interaction network of AFRMICGs. **(C)** Boxplot of expression levels of AFRMICGs. **(D,E)** The **(D)** pathway, **(E)** biological process, cellular component, and molecular function of AFRMICGs. **(F)** The degree of immune cell infiltration for each sample. **(G)** Boxplot showing differences in immune infiltration between AF and control groups. **(H)** Correlation analysis of AFRMICGs and immune cells. ^*^ *P* < 0.05, ^**^ *P* < 0.001, ^***^ *P* < 0.0001.

The database retrieved 2008 genes associated with mitophagy and 2,112 genes relate d to ion channels (Supplementary Table S1). The intersection with DEGs identified 9 AFRMICGs (BAX, CTNNB1, DPYSL2, EPHX1, GLUL, GNB2, MIF, MYC, TLR4), which were all upregulated in AF (Figures 3A–C). Functional enrichment analysis of AFRMICGs identified 21 biological processes, 6 cellular components, 2 molecular functions, and 16 pathways. The biological processes were mainly enriched in the regulation of chemokines, fibroblast proliferation, apoptotic processes, and tumor necrosis factor production; cellular components were related to extracellular exosomes, the cytoplasm, and the cell periphery; molecular functions were related to identical protein binding and transcription coregulator binding; and signaling pathways were related to viral and cellular infections, cancer, necroptosis, and the PI3K-Akt signaling pathway (Figures 3D,E).

To explore the immune infiltration microenvironment in patients with AF, this study analyzed the abundance of immune cells in each individual (Figure 3F). The analysis showed that patients with AF had a higher abundance of resting dendritic cells and neutrophils and a lower abundance of follicular helper T cells, M2 macrophages, and activated dendritic cells (Figure 3G). Correlation analysis showed that AFRMICGs were significantly associated with dendritic cells, mast cells, and NK cells (Figure 3H). The above results suggest that the immune infiltration microenvironment is dysregulated in AF patients, and AFRMICGs may regulate the abundance of immune cell infiltration and consequently affect the immune microenvironment in AF patients.

### 3.3 Machine learning screening of hub genes

**FIGURE 4:**
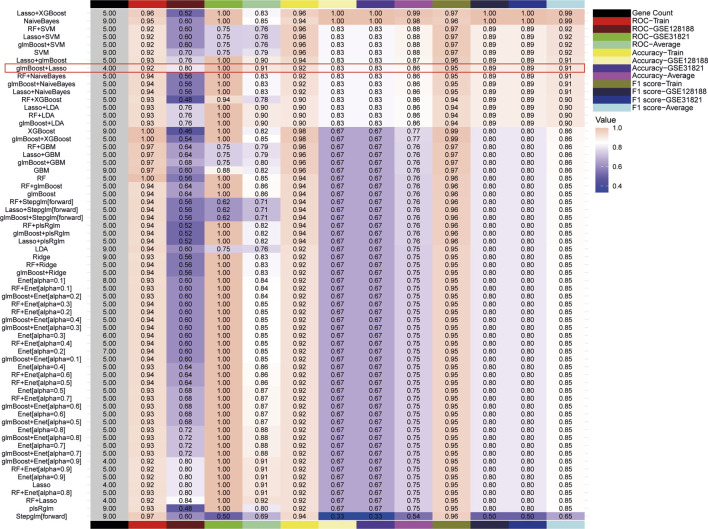
65 machine learning models were screened for Hub genes, and the optimal model was determined based on F1 scores, receiver operating characteristic curves, and accuracy.

To mitigate the risk of overfitting, this study utilized 65 machine learning algorithms to further screen for Hub genes. The top-performing models were identified based on the criteria of ROC curves, accuracy, and F1 scores. When ranking according to the mean F1 scores, the top 10 models were found to be LASSO + XGBoost, NaiveBayes, RF + SVM, glmBoost + SVM, LASSO + SVM, SVM, Lasso + XGBoost, glmBoost + Lasso, RF + NaiveBayes, and glmBoost + NaiveBayes. However, the AUC of the ROC curves for all models, with the exception of glmBoost + Lasso, was less than 0.8 for the validation set GSE128188, indicating suboptimal diagnostic efficacy (Figure 4). Consequently, glmBoost + Lasso was deemed the optimal model, which included four genes: BAX (log_2_FC = 0.617, adj. P = 2.24e-05), GLUL (log_2_FC = 0.880, adj. P = 4.02e-04), MIF (log_2_FC = 0.645, adj. P = 2.72e-04), and TLR4 (log_2_FC = 0.691, adj. P = 4.04e-03).

### 3.4 Disturbed expression of mitophagy and ion channel-related genes in model rats

**FIGURE 5:**
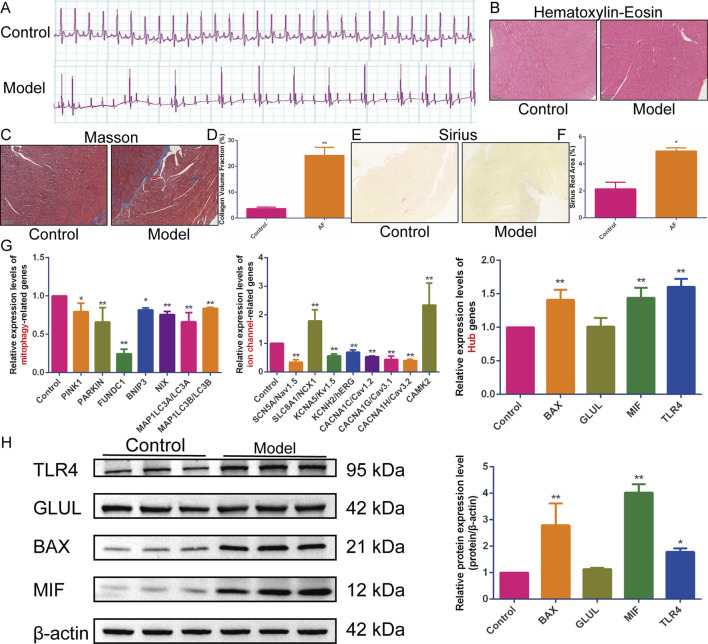
*In vivo* experimental validation. **(A–F)** The **(A)** electrocardiogram, **(B)** hematoxylin-eosin, **(C)** Masson, **(D)** quantification of collagen volume fraction, **(E)** Sirius staining, and **(F)** quantification of Sirius red staining area percentage in rats with acetylcholine-calcium chloride-induced atrial tachyarrhythmias (*n* = 5). **(G)** Quantitative Real-Time Polymerase Chain Reaction (qRT-PCR) to detect the relative expression levels of Hub genes, mitophagy, and ion channel-related genes (*n* = 5). **(H)** Western blotting to detect the expression of BAX, TLR4, MIF, and GLUL proteins (*n* = 3). Results are expressed as the mean ± S.D. ^*^ *P* < 0.05 vs. the control group, ^**^ *P* < 0.01 vs. the control group. The normality of data distribution was assessed using the Shapiro-Wilk test, and the homogeneity of variances was verified by the Brown-Forsythe test. For comparisons among multiple groups that satisfied both normality and homogeneity of variances, a one-way analysis of variance (ANOVA) was employed, followed by Tukey’s honestly significant difference (HSD) post hoc test for specific group comparisons. For data that did not meet the assumptions of parametric tests, the non-parametric Kruskal-Wallis H test was used.

Electrocardiograms from the model group showed the replacement of P waves with rapid, irregular fibrillatory waves (f waves) or flutter waves (F waves), along with an irregularly irregular ventricular response, confirming the induction of atrial tachyarrhythmias (Figure 5A). Quantitative analysis revealed that the induced arrhythmia was characterized by a significantly higher atrial rate (886.2 ± 87.64 bpm, *n* = 5) compared to the sinus rhythm in control rats (412 ± 23.49 bpm, *n* = 5, P < 0.001). Pathological staining revealed disordered cardiomyocyte arrangement, collagen fiber proliferation, widened interstitium, fibrous septa formation, uneven cytoplasmic staining, and inflammatory cell infiltration in the model group (Figures 5B–F).

Subsequently, the expression levels of sodium channel genes (SCN5A/Nav1.5, SLC8A1/NCX1), potassium channel genes (KCNA5/Kv1.5, KCNH2/hERG), calcium channel genes (CACNA1C/Cav1.2, CACNA1G/Cav3.1, CACNA1H/Cav3.2, CAMK2/CaMKII), mitophagy-related genes (PINK1, Parkin, FUNDC1, BNIP3, NIX, LC3II/MAP1LC3B, LC3I/MAP1LC3A), and Hub genes (BAX, GLUL, MIF, TLR4) were detected in model rat hearts. qRT-PCR results showed that, compared with the control group, the expression of BAX, MIF, TLR4, SLC8A1/NCX1, and CAMK2/CaMKII was upregulated in the myocardial tissue of model rats. In contrast, the expression of SCN5A/Nav1.5, KCNA5/Kv1.5, KCNH2/hERG, CACNA1C/Cav1.2, CACNA1G/Cav3.1, CACNA1H/Cav3.2, PINK1, Parkin, FUNDC1, BNIP3, NIX, LC3I/MAP1LC3A, and LC3II/MAP1LC3B was downregulated (Figure 5G). Western blotting analysis further confirmed the upregulation of BAX, MIF, and TLR4 protein expression in the cardiac tissue of model rats. However, the expression of the GLUL gene and protein was not significantly altered (Figure 5H). These results indicate significant myocardial histopathological changes and fibrosis in model rats, accompanied by disturbed expression of genes related to mitophagy and sodium-potassium-calcium ion channels.

### 3.5 Construction of nomogram, “TFs-mRNA”, and “lncRNA-miRNA-mRNA” ceRNA networks

**FIGURE 6:**
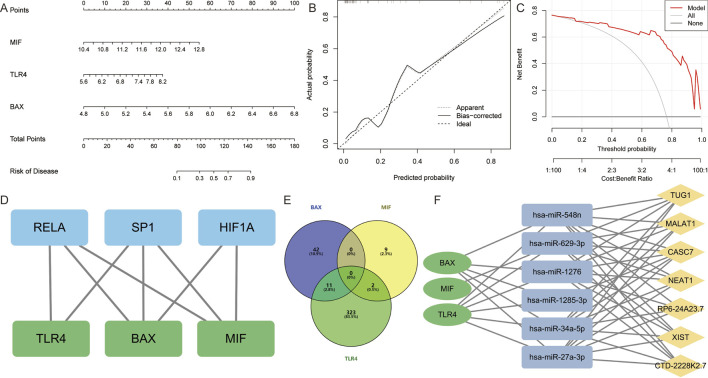
Construction and assessment of nomogram. **(A)** A nomogram was constructed based on BAX, TLR4, and MIF to predict the risk of developing AF. **(B,C)** Construction of calibration curve and decision curve for assessing the predictive efficiency of the nomogram. **(D)** Construction of “Transcription Factor-Hub Genes” network. **(E)** Venn diagram showing miRNAs predicted by Hub genes. **(F)** Construction of “lncRNA-miRNA-mRNA” ceRNA interaction network.

The experimental results indicated that the expression of BAX, MIF, and TLR4 genes and proteins was upregulated in the atrial myocardial tissue of model rats, while GLUL did not show significant changes. Therefore, BAX, MIF, and TLR4 are considered core AFRMICGs. Subsequently, a nomogram was constructed based on the Hub genes (Figure 6A). Both decision curve analysis and calibration curve assessment indicated satisfactory diagnostic performance, showing that the model could effectively distinguish between AF and normal samples (Figures 6B,C).

The TRRUST database identified 29 TFs for BAX, 5 TFs for MIF, and 6 TFs for TLR4. The hTFtarget database predicted 176 TFs for BAX, 160 TFs for MIF, and 65 TFs for TLR4. Integration of these results indicated that SP1 and RELA are common TFs regulating the Hub genes. Additionally, HIF1A was identified as a common TF for BAX and MIF (Figure 6D).

The database predicted 53 miRNAs for BAX, 11 for MIF, and 336 for TLR4. It identified hsa-miR-27a-3p, hsa-miR-34a-5p, hsa-miR-4524a-3p, hsa-miR-1276, hsa-miR-3148, hsa-miR-1285-3p, hsa-miR-3187-5p, hsa-miR-4695-5p, hsa-miR-5685, hsa-miR-4690-3p, and hsa-miR-548n as miRNAs targeting BAX and TLR4. Additionally, hsa-miR-629-3p and hsa-miR-4713-5p were found to target both MIF and TLR4 (Figure 6E). Further analysis revealed that CASC7, CTD-2228K2.7, MALAT1, NEAT1, RP6-24A23.7, TUG1, and XIST are upstream lncRNAs for hsa-miR-27a-3p, hsa-miR-34a-5p, hsa-miR-1276, hsa-miR-548n, and hsa-miR-1285-3p. Additionally, CASC7 was identified as an upstream lncRNA of hsa-miR-1285-3p (Figure 6F).

## 4 Discussion

AF remains a major global public health problem, imposing a significant treatment burden due to limited therapeutic options. Therefore, elucidating the pathogenesis of AF to identify precise intervention targets is crucial for improving its management. This study used a bioinformatics approach focusing on mitophagy and ion channels to explore their key genes and potential mechanisms in AF. This study first identified the DEGs in AF and investigated the potential pathogenesis of AF through functional enrichment analysis. The results revealed that the pathogenesis of AF is closely related to inflammation, immune response, ion channels, and various organelles.

Structural and electrical remodeling are currently considered the main pathogenic mechanisms of AF. However, recent studies have highlighted that inflammation and immune response also play significant roles in the development and progression of AF. Inflammation and its associated immune responses have been implicated in the triggering and sustenance of AF episodes. Inflammation-induced AF is perpetuated through a cycle in which it stimulates new inflammatory responses and initiates atrial remodeling. Mediators of the inflammatory response increase susceptibility to AF by altering atrial electrophysiology and structural substrates. Inflammation also disrupts calcium homeostasis and connexin function, thereby increasing triggers for heterogeneous atrial conduction. Additionally, inflammation mediates the activation of fibrotic pathways via fibroblasts, transforming growth factor-β, and matrix metalloproteinases, which promotes myocardial fibrosis (Hu et al., 2015; Harada and Nattel, 2021). A meta-analysis has shown that inflammatory cytokine levels in perioperative patients are significantly associated with the development of postoperative AF (Weymann et al., 2018). Elevated expression of inflammatory factors, such as C-reactive protein, interleukin-6, interleukin-2, interleukin-1β, and tumor necrosis factor-α, has been significantly and positively correlated with the incidence of AF (Hu et al., 2015; Harada and Nattel, 2021). The activation of NLRP3 inflammasomes is observed in cardiomyocytes from patients with AF, contributing to AF by recruiting immune cells and enhancing atrial structural and electrical remodeling39. Immune infiltration analysis in this study revealed significant dysregulation of the immune microenvironment in the left atrial tissues of patients with AF. Specifically, a high level of infiltration of neutrophils and a lower abundance of anti-inflammatory M2 macrophages were observed in the atrial myocardium of these patients. These findings suggest the presence of a chronic inflammatory state in AF patients. Immune cells, including T cells, macrophages, and neutrophils, mediate cardiac inflammation by infiltrating the myocardium from peripheral tissues and inducing localized inflammatory responses (Yao et al., 2018; Li and Brundel, 2020; Yao et al., 2022). In conclusion, inflammation and immune response play a crucial role in the pathogenesis of AF. However, the exact mechanisms through which these processes contribute to the development and progression of AF remain incompletely defined and require further investigation.

Ion channel abnormalities are equally important in the pathogenesis of AF. In the normal cardiac cycle, electrical impulses originating from the sinoatrial node propagate through the atria, atrioventricular node, His bundle, and Purkinje fibers to trigger synchronized ventricular contraction. Myocardial action potentials depend on sequential activation/inactivation of depolarizing (Na^+^, Ca^2+^) and repolarizing (K^+^) ion channels. During depolarization, Ca^2+^ influx via voltage-gated channels triggers RyR2-mediated calcium-induced calcium release (CICR) from the SR. This calcium binds troponin C, initiating cardiomyocyte contraction. Diastole requires membrane repolarization (driven by Kv and Kir channels) and calcium clearance via SERCA and NCX10, (Roden et al., 2002; Heijman et al., 2014). *In vivo* experiments in this study revealed significant dysregulation of ion channel gene expression in the Ach-CaCl_2_-induced rat model of AF. Specifically, sodium channel (SCN5A/Nav1.5), potassium channel (KCNA5/Kv1.5, KCNH2/hERG), and calcium channel (CACNA1C/Cav1.2, CACNA1G/Cav3.1, CACNA1H/Cav3.2) gene expression was significantly downregulated, while SLC8A1/NCX1 and CAMK2/CaMKII gene expression was significantly upregulated. Sodium channel SCN5A (Nav1.5) downregulation reduces the phase 0 depolarization rate and conduction velocity. Potassium channel (KCNA5/Kv1.5, KCNH2/hERG) downregulation prolongs action potential duration and increases repolarization dispersion. Calcium channel (CACNA1C/Cav1.2, CACNA1G/Cav3.1, CACNA1H/Cav3.2) downregulation shortens action potential duration. Upregulated SLC8A1 (NCX1) generates net inward currents, delaying repolarization and increasing electrical instability. Activated CAMK2/CaMKII exacerbates calcium overload (Roden et al., 2002; Heijman et al., 2014). These findings suggest that atrial myocytes in the Ach-CaCl_2_-induced rat model of AF undergo typical processes of electrical remodeling and calcium-regulated remodeling. Electrical remodeling is characterized by downregulation of sodium, potassium, and calcium channels, leading to conduction slowing, prolonged action potential duration, and repolarization dissociation. Calcium-regulated remodeling involves upregulation of NCX1, activation of CaMKII, calcium overload, calcium leakage, arrhythmogenic inward currents, and delayed afterdepolarizations.

Mitochondrial dysfunction is a key mechanism driving electrical remodeling, as it promotes AF episodes by impairing cardiomyocyte ion channel function and expression through energy deficiency and ROS bursts. Studies have confirmed that targeting mitochondrial function can effectively alleviate AF (Ge et al., 2025). Mitophagy, a key component of mitochondrial quality control, helps to improve overall mitochondrial function by removing dysfunctional mitochondria. The key proteins of the ubiquitin-dependent pathway are PINK1 and Parkin, constituting PINK1/Parkin-mediated mitophagy. PINK1, a kinase on the mitochondrial outer membrane, is usually degraded after translocation to the inner membrane. When mitochondria are damaged and depolarized, PINK1 accumulates on the outer membrane, dimerizes, and autophosphorylates to activate. Activated PINK1 phosphorylates Parkin, activating its E3 ligase activity to ubiquitinate mitochondrial proteins. This process facilitates the recognition and clearance of damaged mitochondria. In contrast, non-ubiquitin-dependent mitophagy is mediated by receptors like NIX, BNIP3, and FUNDC1 on the mitochondrial outer membrane. Upon damage, these receptors bind to LC3II via their LC3-binding domains, thereby anchoring damaged mitochondria to the autophagosomal membrane and initiating mitophagy (D'Arcy, 2024; Lu et al., 2023). This study found that PINK1, Parkin, FUNDC1, BNIP3, NIX, LC3I/MAP1LC3A, and LC3II/MAP1LC3B genes were downregulated in the Ach-CaCl_2_-induced rat model, suggesting a significant impairment of mitophagy. Therefore, moderate activation of mitophagy is considered a potential therapeutic mechanism for AF.

Functional enrichment analysis of AFRMICGs revealed that the PI3K/AKT signaling pathway is a key regulator linking mitophagy to ion channel function in AF. The PI3K/AKT signaling pathway is a critical cellular pathway that modulates a wide range of biological processes, including cell growth, proliferation, survival, metabolism, migration, and apoptosis. The role of the PI3K/AKT pathway in AF has been extensively studied, yet its specific effects appear to vary considerably across different experimental models and species. For instance, in several animal models of AF, such as heart failure-induced AF in rats (Chong et al., 2015) and rabbits (Liu et al., 2016), the spontaneously hypertensive rat model (Wang et al., 2015), the canine model of rapid atrial pacing (Zhao et al., 2021), the diabetes-induced AF rat model (Xue et al., 2020), and the Ach-CaCl_2_-induced rat model (Xu et al., 2024; Zhang et al., 2025a), the expression of PI3K and AKT was significantly downregulated. Conversely, in the Angiotensin II (Ang II)-induced AF mouse model, the expression of PI3K and AKT was significantly upregulated (Li et al., 2025; Chen et al., 2024; Ji and Ning, 2024). A finding by Badreldin et al., which reported upregulation of PI3K and AKT in an Ach-CaCl_2_-induced rat model, is inconsistent with the majority of previous studies (Badreld et al., 2024).

The role of the PI3K/AKT signaling pathway in regulating mitochondrial function, autophagy, and ion channels, such as calcium and sodium channels, has been partially elucidated in the context of AF. For instance, Xu et al. demonstrated that activation of the PI3K/AKT signaling pathway improved mitochondrial function in the Ach-CaCl_2_-induced rat model. This activation restored the activity of Ca^2+^-ATPase and downregulated the expression of p-CaMKII/CaMKII, p-RyR2/RyR2, and NCX1, while upregulating SERCA2 and Cav1.2 expression (Xu et al., 2024). In another study, ibrutinib-treated HL-1 cells exhibited increased susceptibility to AF due to the inhibition of the PI3K/AKT/mTOR signaling pathway, which enhanced autophagy and led to connexin degradation (Qin et al., 2024). Additionally, the PI3K/AKT signaling pathway was shown to prevent cellular remodeling in rapidly paced HL-1 atrial myocytes by inhibiting late sodium currents (Ko et al., 2023). Moreover, laminar shear stress was found to upregulate KCa2.3 expression in H9c2 cardiomyocytes through activation of the PI3K/AKT/p300 pathway, thereby affecting potassium-calcium channel function (Li et al., 2019). In a rabbit model of heart failure-induced AF, activation of the PI3K/AKT pathway led to upregulation of ion channels such as KvLQT1, Cav1.2, and SERCA2a, which improved atrial electrical remodeling and reduced fibrosis (Liu et al., 2016).

Subsequently, 65 machine learning models were constructed, among which glmBoost + Lasso was confirmed as the optimal model based on ROC curves, accuracy, and F1 scores. Through *in vivo* experimental validation, BAX, MIF, and TLR4 were ultimately identified as Hub genes for AF.

BCL-2-associated X protein (BAX), a key pro-apoptotic member of the BCL-2 family, significantly contributes to the pathophysiology of AF. Studies have demonstrated that BAX expression is markedly upregulated in AF patients and animal models, including rats and dogs (Li et al., 2018; Jia et al., 2017; Yuan et al., 2021). BAX levels correlate positively with the severity of AF, with higher levels observed in permanent AF compared to persistent and paroxysmal AF in patients with rheumatic heart disease and in persistent AF than in paroxysmal AF (Diao et al., 2016). Furthermore, elevated BAX expression in a senescent AF model suggests its potential as a biomarker for aging-related AF (Xu et al., 2013). BAX’s interaction with mitophagy primarily involves the regulation of apoptosis. Upon apoptotic stimulation, BAX accumulates on the outer mitochondrial membrane, forming oligomers that enhance mitochondrial outer membrane permeability, leading to the release of pro-apoptotic factors such as cytochrome c, thereby initiating apoptosis. This mitochondrial outer membrane permeability-mediated mitochondrial swelling exposes the inner mitochondrial membrane, activating mitophagy via a PINK1-Parkin-independent pathway (Saunders et al., 2024). While this process may serve as a compensatory mechanism for clearing damaged mitochondria, sustained high BAX expression can disrupt the balance between autophagy and apoptosis. Additionally, BAX may play a role in AF electrical remodeling by affecting calcium cycling. It has been shown to trigger endoplasmic reticulum ryanodine receptors (RyRs), facilitating the release of Ca^2+^ from the endoplasmic reticulum into the mitochondrion (SanMartín et al., 2020). Conversely, BAX deficiency results in decreased mitochondrial Ca^2+^ uptake, highlighting BAX as a crucial regulator of calcium homeostasis (Scorrano et al., 2003).

Macrophage Inflammation Factor (MIF) is a pro-inflammatory cytokine released by activated macrophages, which has been shown to be elevated in patients with AF and correlates with disease severity. Specifically, MIF levels are highest in patients with permanent AF, followed by those with persistent AF, and lowest in those with paroxysmal AF (Wan and Li, 2018; Li et al., 2020). Mendelian randomization analysis has confirmed a causal link between MIF and AF (Wei et al., 2023). MIF modulates calcium homeostasis and ionic currents by affecting L-type and T-type calcium channels (Rao et al., 2013; Rao et al., 2009). In HL-1 cardiomyocytes, an *in vitro* model, MIF treatment induced enhanced calcium transients, increased SR calcium content, elevated sodium/calcium exchanger efflux rate, calcium leak, transient outward potassium current, and ultra-rapid delayed rectifier potassium current (Cheng et al., 2020). MIF induces abnormal sodium and calcium regulation through activation of the CaMKII signaling pathway and ROS, which contributes to the triggering of AF during inflammation (Chin et al., 2023). MIF also inhibits mitophagy; it has been found that loss of autophagy/mitophagy leads to the accumulation of cytoplasmic ROS and mitochondrial DNA, which activates immune signaling pathways and ultimately leads to the release of MIF (Harris et al., 2018). In a septic kidney injury model, MIF inhibits mitophagy by disrupting the PINK1-Parkin interaction (Li et al., 2024a). However, another study shows that MIF acts as a protective factor against myocardial contractile dysfunction caused by side-stream smoke exposure by promoting mitophagy and the formation of autophagolysosome (Wang et al., 2020). Additionally, MIF appears to exert a certain improvement effect in myocardial fibrosis (Zhao et al., 2025; Zhu et al., 2021). Thus, MIF plays a pivotal role in AF pathogenesis, influencing inflammation, mitophagy, ion channels, and myocardial fibrosis. Targeting MIF and its signaling pathways may offer therapeutic strategies for AF management, potentially alleviating symptoms and improving patient outcomes.

Toll-like Receptor 4 (TLR4), a member of the Toll-like Receptor family and a Pattern Recognition Receptor within the human immune system, has been linked to AF. Studies have reported elevated TLR4 expression in AF patients (Gur et al., 2018; Gurses et al., 2016), with particularly high levels observed in those with persistent AF compared to paroxysmal AF, indicating a potential correlation between TLR4 and AF severity81. TLR4 is emerging as a potential biomarker for new-onset AF following acute myocardial infarction, with significantly increased levels observed in patients experiencing new-onset AF compared to healthy controls and those with myocardial infarction alone (Zhang et al., 2018). In hypertensive rats, silencing TLR4 expression has been shown to reduce left atrial fibrosis and susceptibility to AF (Ge et al., 2022). Furthermore, MD1, a negative regulator of TLR4, when deleted, enhances AF susceptibility and induces atrial remodeling in diabetic cardiomyopathy mice through activation of the TLR4/NF-κB signaling pathway (Shi et al., 2023). TLR4 plays a crucial role in regulating intracellular ionic homeostasis and currents by influencing sodium (Li et al., 2024b), potassium (Bowen et al., 2024), L-type and T-type calcium channels (Li et al., 2017; Zhang et al., 2025b), and calcium homeostasis (Zhu et al., 2024). In a high-fat diet-induced AF model, MD1 knockdown promotes atrial remodeling in AF mice by activating the TLR4 signaling pathway, which modulates the expression of CaMKII, MyD88, and NF-κB, thereby affecting the expression of Kv4.2, Kv4.3, Kv1.5, Kv2.1, and Cav1.2 channel proteins, and regulates potassium-calcium channels (Shuai et al., 2019b; Shuai et al., 2019a). HMGB1 disrupts excitation-contraction coupling in cardiomyocytes by increasing SR Ca2+ leak via the TLR4-ROS signaling pathway (Zhang et al., 2014). TLR4 has been implicated in the regulation of mitophagy in conditions such as atherosclerosis (Chang et al., 2025), heat stroke-induced brain injury (Lin et al., 2024b), and acute pancreatitis-associated splenic injury (Wen et al., 2022), although its specific role in AF is not fully understood. Furthermore, TLR4-mediated inflammatory responses are crucial in regulating myocardial hypertrophic remodeling (Xiao et al., 2020). Studies have shown that cardiac-specific deletion of TLR4 reduces the severity of Ang II-induced myocardial fibrosis (Theobald et al., 2023). This suggests that modulating the TLR4 signaling pathway could be an effective strategy for managing the pathophysiology of cardiac hypertrophy.

This study employed integrated bioinformatics analyses to identify key genes and mechanisms linking mitophagy and ion channel dysfunction in AF. This study’s results establish the PI3K/AKT signaling pathway and genes BAX, TLR4, GLUL, and MIF as central regulators (Hub genes). Validation in an Ach-CaCl_2_-induced rat model confirmed dysregulation of mitophagy and sodium/potassium/calcium channel-related genes in atrial tissue, with BAX, TLR4, and MIF further identified as core AFRMICG Hub genes. Upstream regulatory networks (transcription factors, ceRNA interactions) for these Hub genes were also explored using public databases. These findings provide valuable mechanistic insights into AF pathogenesis and highlight potential therapeutic targets.

However, this study has several limitations. First, to address the heterogeneity across AF datasets, the analyses were restricted to training sets from a single platform (GPL570) and region (China: GSE41177, GSE79768). This approach may introduce population bias and limit the generalizability of the findings to other populations. Second, only expression differences of the Hub genes were validated in rats, without further functional investigation. Furthermore, the use of exclusively male animals in the experimental validation, though intended to minimize variability from the estrous cycle, represents a significant limitation. This design precludes assessment of potential sex-dependent differences in the mitophagy-ion channel axis, thus limiting the applicability of the results to females. Previous studies have shown that AF patients of different genders exhibit distinct biomarkers (Wu et al., 2024). Additionally, the isoflurane anesthesia used during tissue collection in rats, while standard, is known to modulate various cardiac ion channels. Its potential influence on the electrophysiological state at the time of sampling cannot be entirely ruled out, which may confound the interpretation of ion channel-related findings. Therefore, the changes in these genes in human AF patients, particularly across different sexes, require further validation to confirm their broad clinical relevance. Moreover, the study lacks gain- and loss-of-function experiments to elucidate the causal roles of BAX, MIF, and TLR4 in regulating mitophagy and ion channels. Finally, while the study implicates a mitophagy-ion channel axis, the specific molecular interactions and functional consequences require experimental validation.

In addition, it is important to note a limitation regarding the *in vivo* model. The Ach-CaCl_2_ induction method can produce a spectrum of atrial tachyarrhythmias. While electrocardiogram patterns consistent with AF were observed, characterized by irregular f waves and variable R-R intervals, some recorded episodes also displayed electrocardiographic features of atrial flutter. Both AF and atrial flutter share common pathophysiological substrates, such as electrical and structural remodeling. Therefore, the key findings of this study regarding the dysregulation of mitophagy and ion channels are interpreted in the context of a sustained, rapid atrial tachyarrhythmia model, which robustly recapitulates critical aspects of the clinical arrhythmogenic substrate.

To address these limitations, future research should expand the datasets to include male and female subjects from diverse ethnic and geographic backgrounds, enabling the development of more comprehensive and generalizable models of AF mechanisms. Subsequent studies should conduct sex-stratified analyses to systematically explore potential differences in gene expression, pathway activity, and immune infiltration between sexes. Additionally, gain- and loss-of-function experiments *in vitro* and in animal models are essential to establish the causal roles of the identified hub genes (BAX, MIF, TLR4) and to dissect the functional interplay within the mitophagy-ion channel axis. Finally, future work should explicitly address the impact and biological implications of sex differences, emphasizing this as a key factor for understanding AF pathophysiology and developing personalized therapeutic strategies.

## 5 Conclusion

BAX, MIF, and TLR4 are key genes linking mitophagy and ion channels in AF, which appear to influence the immune microenvironment by modulating immune cell infiltration.

## Data Availability

The original contributions presented in the study are included in the article/Supplementary Material, further inquiries can be directed to the corresponding author.
